# The TNFα-binding domain of the therapeutic antibody adalimumab elicits CD4 T-cell responses in rheumatoid arthritis patients

**DOI:** 10.3389/fimmu.2025.1549781

**Published:** 2025-07-04

**Authors:** Mateusz Makuch, Josine van Beek, Carla A. Wijbrandts, Marja Aalbers, Philippe Stas, Alexander B. Meijer, Anja ten Brinke, Theo Rispens, Paul Peter Tak, Gertjan Wolbink, Janine Schuurman, Paul W. H. I. Parren, S. Marieke van Ham

**Affiliations:** ^1^ Sanquin Research, Department of Immunopathology, and Landsteiner Laboratory, Amsterdam University Medical Center (UMC), University of Amsterdam, Amsterdam, Netherlands; ^2^ Centre for Immunology of Infectious Diseases and Vaccines, National Institute for Public Health and the Environment (RIVM), Bilthoven, Netherlands; ^3^ Department of Rheumatology, Reade, Amsterdam, Netherlands; ^4^ ImmunXperts, Gosselies, Belgium; ^5^ Department of Plasma Proteins, Van Creveld Laboratory of University Medical Center (UMC) Utrecht and Sanquin Research, Amsterdam, Netherlands; ^6^ Department of Rheumatology and Clinical Immunology, Amsterdam Rheumatology and Immunology Center (ARC), Amsterdam, Netherlands; ^7^ Candel Therapeutics, Needham, MA, United States; ^8^ Genmab, Utrecht, Netherlands; ^9^ Department of Immunology, Leiden University Medical Center, Leiden, Netherlands; ^10^ Swammerdam Institute for Life Sciences, University of Amsterdam, Amsterdam, Netherlands

**Keywords:** anti-drug antibodies, anti-TNF therapy, immunogenicity, rheumatoid arthritis, CD4 T cells

## Abstract

Treatment efficacy of patients receiving anti-TNF antibodies is limited by the formation of anti-drug antibodies. These are observed in most adalimumab-treated rheumatoid arthritis patients, despite the adjuvant-free and human sequence-derived nature of the antibody. The class switched phenotype and high affinity of these antibodies suggest CD4 T-cell involvement in their formation. In this study, we investigated the potential epitopes in the functional domain of adalimumab and assessed their actual HLA II presentation and induction of CD4 T-cell responses in exposed patients. The binding strength of overlapping adalimumab-derived peptides to 27 DR and 14 DQ HLA alleles was predicted *in silico*. 10 strong and 44 medium-binding 10-mer peptides were identified within the variable regions of the heavy and light chain of adalimumab. HLA-DR-mediated antigen presentation of selected peptides by monocyte-derived dendritic cells was determined by mass spectrometry of the peptide pool eluted from isolated HLA-DR complexes. Binding of the variable region peptides of heavy (H41-62) and light chains (L18-39) was demonstrated. The presence of adalimumab-specific CD4 T-cells in adalimumab-experienced patients was investigated via peptide stimulation of peripheral blood mononuclear cells and assessment of T-cell proliferation. Anti-adalimumab CD4 T-cell responses were observed against four variable region peptides in a group of adalimumab-experienced RA patients. Some of these responses were also present in healthy control donors. This study identifies immunologically relevant CD4 T-cell epitopes in the variable region of the human therapeutic antibody adalimumab based on RA patients’ reactivity. Modification of these epitopes or concomitant therapy that targets or prevents adalimumab-specific T cell responses could be beneficial for patients with significant anti-drug responses.

The introduction of TNF-α inhibitor therapies has been a breakthrough in treatment of patients with immune-mediated inflammatory disorders (1), after TNF was recognized as a driving force in the pathology of many of these conditions (2–4). To date, several TNF antagonists are available for patients, including infliximab, etanercept, adalimumab, certolizumab pegol and golimumab. These biologicals are widely used because of their compelling benefit in treatment of rheumatoid arthritis (RA), ankylosing spondylitis, psoriatic arthritis, and other immune-mediated disorders (1, 5). Substantial numbers of patients however, show no clinical response or lose their initial responsiveness upon prolonged treatment (5–10). Studies on chimeric infliximab and fully human adalimumab by us and others have associated this lack of responsiveness in part with decreased plasma levels of the therapeutic antibody and development of anti-drug antibodies (ADA) (5, 11–16). In adalimumab-treated patients the anti-drug response varies between 5% and 44% (12, 17–19) and is similar to that of infliximab, where 8% to 52% of RA patients are reported to form antibodies against the drug (8, 11, 17, 20, 21). Golimumab, which was derived from genetically modified mice carrying human immunoglobulin genes, induced ADA in up to 22% of RA patients co-treated with methotrexate (MTX) during 68 weeks follow-up (22). These findings demonstrated that immunogenicity of therapeutic antibodies does not directly reflect their humanization level (23).

We have shown that the majority of ADA found in adalimumab-treated patients is directed against the idiotype – antigen-binding part of an antibody (15, 16). The observed induction of the IgG subclass of anti-adalimumab antibodies (AAA), their high binding affinity together with high somatic hypermutation frequency in isolated antibody sequences all strongly point to a CD4 T cell dependent origin of the anti-adalimumab response. Indeed, a substantial immunogenic potential to induce CD4 T cell responses was observed in complementarity determining regions (CDR) of therapeutic antibodies (24). The presence of a pre-existing low-frequency CD4 T cell repertoire against undefined epitopes in adalimumab was shown *in vitro* in the naïve T cell pool of healthy donors (25, 26). This indicates that adalimumab has the potential to generate CD4 T cell responses *in vivo*. This was further expanded upon using peptide-based approaches in MAPPS (MHC-associated peptide proteomics) assays (27, 28), albeit again depending on healthy donor- derived APCs and T cells. In another study, the development of the anti-drug responses was attributed to a potential cross-reactivity with influenza hemagglutinin and identified heavy chain region 95–109 and 97–111 as potential T and B cell epitopes mimicking those present in HA (29). If this prediction was accurate, it would suggest a pre-existing cross-reactive memory CD4 T cell compartment that underlies anti-drug antibody formation. So far however, no reported data demonstrate the existence of CD4 T cell responses against defined epitopes of adalimumab in those treated with adalimumab and the overlap between patient T cell epitopes and those identified in healthy donors remains unexplored.

In this study, we set out to identify and validate *in silico* predicted HLA class II-binding epitopes in adalimumab and elucidate whether these epitopes elicit CD4 T cell reactivity in treated RA patients. We established that the most epitope-dense parts of adalimumab are located within the variable regions of the heavy and light chains of the therapeutic antibody. We show that predicted epitopes can bind to HLA class II on dendritic cells and demonstrate that CD4 T cell reactivity against variable epitopes of adalimumab occurs both in adalimumab-treated RA patients and in healthy donors, with a significant overlap between the two groups, adding to the evidence that anti-drug reactivity can be associated with pre-existing T cell repertoires.

## Results

### Multiple CD4 T cell epitopes are predicted *in silico* within the adalimumab sequence

To identify potential HLA class II binding peptides that may elicit CD4 T cell responses against adalimumab, the sequences of the heavy and light chains of the therapeutic antibody were subjected to *in silico* analyses using the Epibase™ prediction tool. **Table 1** shows the number of 10-mer peptides binding with a predicted strong or medium affinity to the HLA-DR, DQ or DP of a given allotype, calculated separately for each domain of the heavy and light chain of adalimumab. The analysis indicates the presence of potential HLA-DR and HLA-DQ binding epitopes all over the adalimumab heavy and light chain sequences (**Table 1**, first value), whereby the variable regions show the highest epitope-density. The total number of strong and medium binding 10-mer peptides differs between the different HLA molecules. For HLA-DP no strong binding peptides were predicted. Since HLA-DP is expressed at a low level (30, 31) and the most common HLA-DP alleles seem to share a similar specificity (32), we excluded it from further analyses.

**Table 1:** Summary of T cell epitope profiling of adalimumab with Epibase™. 
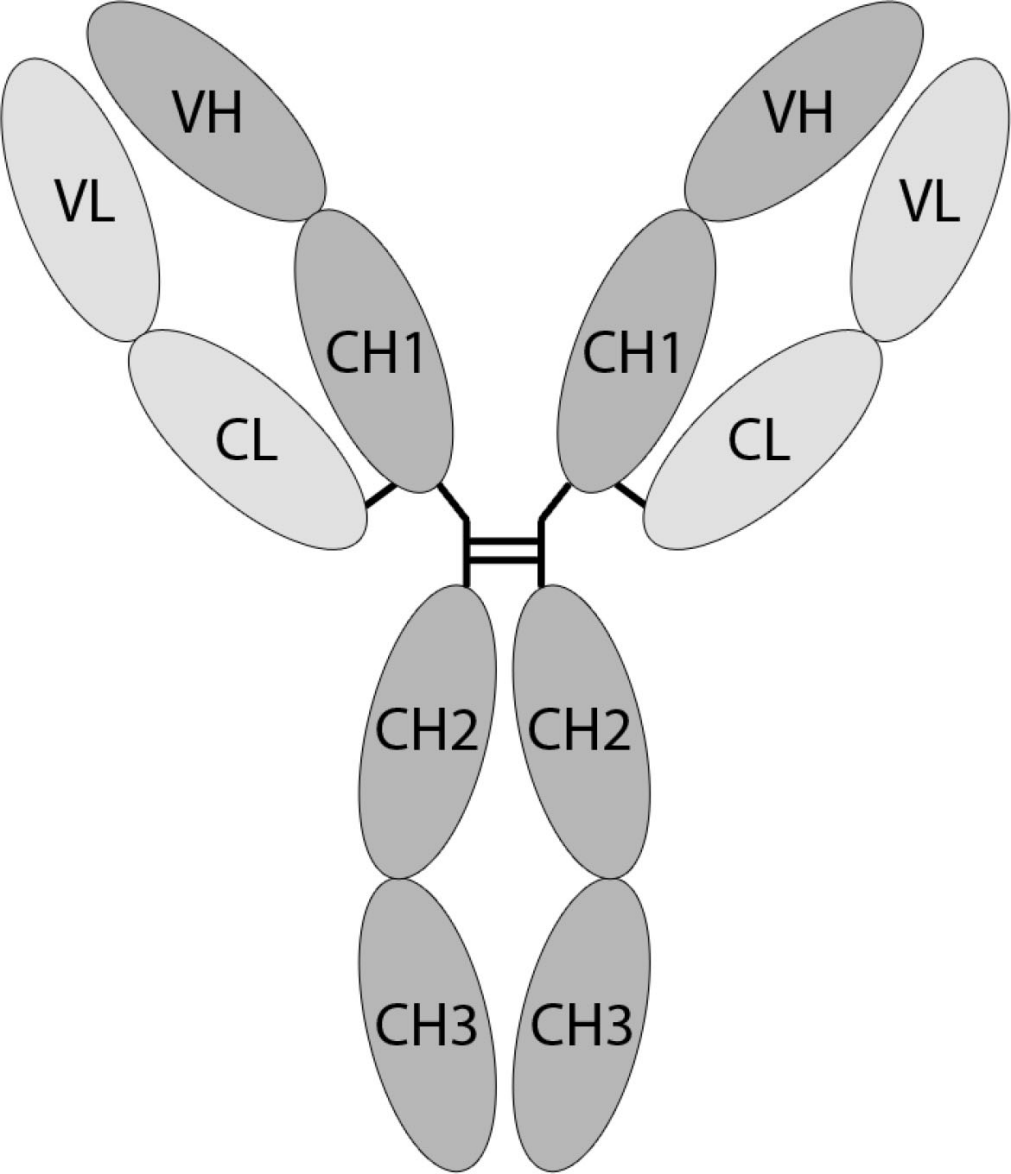

Antibody region	DRB1		DRB 3/4/5		DQ		DP	
	Strong	Medium	Strong	Medium	Strong	Medium	Strong	Medium
VL	8→2	24→11	2→1	7→2	4→1	13→5	0	9→3
CL	4→0	16→0	0	5→0	0	8→0	0	7→0
VH	8→4	28→11	2→1	7→4	2→1	22→11	0	5→2
CH1	6→0	13→0	0	4→0	0	10→0	0	2→0
Hinge	0	0	0	0	0	0	0	0
CH2	5→0	25→0	0	7→0	1→0	11→0	0	5→0
CH3	1→0	21→0	2→0	4→0	2→0	8→0	0	7→0
								
Total	32→6	127→22	6→2	34→6	9→2	72→16	0	35→5

The number of predicted strong or medium-binding CD4 T cell peptides are presented per HLA class II gene family for different parts of the heavy (VH, CH) and light chain (VL, CL) of adalimumab, and for the entire protein. VH/VL, variable domain of heavy/light chain; CH/CL, constant domain of heavy/light chain. Peptides binding to multiple HLAs of the same group are counted as one. Germline filtering eliminates sequences that are common across alleles and therefore likely considered self (pre- and post-arrow values).

The results indicated the presence of potential HLA-DR and HLA-DQ binding epitopes, which were distributed all over the adalimumab heavy and light chain sequences. To select for epitopes that may elicit CD4 T cell reactivity, we considered the T cell responses in patients receiving adalimumab to most likely occur against epitopes of the therapeutic antibody that are different from those present in the antibody germline. Therefore, the epitopes were filtered against a database of germline sequences to exclude the shared antibody sequences. Since every peptide that underwent substitution is a potential T-cell epitope, sequences that contained at least 1 different amino acid compared to germline were considered non-germline. Germline filtering (**Table 1**, second value) reduced the number of potential HLA II epitopes and restricted their presence to epitopes that showed an overlap with parts of the variable regions of adalimumab, with both heavy and light chains containing potential strong and medium binding peptides.

Most predicted peptides bind to multiple HLA alleles. Only DRB1*11:04 and DRB3*03:01 were not predicted to bind adalimumab peptides with strong or medium affinity (not shown). Together, these data show that the variable regions of adalimumab that are most likely to elicit immunity contain CD4 T cell epitopes with predicted high and medium affinity binding to majority of HLA class II alleles. As the prediction tools are heavily reliant on the HLA-binding in the prediction model, it is expected that some of the peptides will fail to show the predicted characteristics.

### *In silico* prediction yields true binding peptides

Epibase™ predicts the strength of HLA class II – peptide binding, but does not prove that those peptides can be presented on the cell surface. Therefore, we investigated whether selected Epibase™-predicted HLA II peptides could indeed bind to HLA class II of antigen presenting cells (APC). Five healthy donors were HLA-typed and APC were tested for binding of peptides derived from the CDR-H2 (H41-62) and CDR-L1 (L18-39) regions of adalimumab. These peptides were chosen, as these were predicted to bind with medium to high affinity to the HLA spectrum of the donor’s multiple HLA types (**Table 2**).

**Table 2:** Summary of HLA allotypes and peptide binding predictions.

Sample		HLA-DR	HLA-DQ	H41-62		H47-68		H88-109		L18-39	
				DR	DQ	DR	DQ	DR	DQ	DR	DQ
RA	1	DRB1*04:01, *16:02*; DRB4*01:03	DQA*01:02/03; DQB*03:02/05:02	1/0	0/1	1/0	0/0	0/2	0/4	0/1	0/2
	2	DRB1*04:01, 07:01; DRB4*01:03	DQA*02:01/03; DQB*03:02/03:03	2/0	1/2	1/0	1/1	0/2	0/4	0/2	0/1
	3	DRB1*07:01, 13:02; DRB3*03:01; DRB4*01:03	DQA*01:02/02:01; DQB*02/06:04	1/1	0/2	0/0	0/1	0/1	0/3	0/2	0/1
	4	DRB1*13:02, 15:01; DRB3*03:01; DRB5*01:01	DQA*01:02; DQB*06:02/06:04	0/2	0/0	0/0	0/0	1/1	0/2	0/3	0/0
	5	DRB1*04:01, 16:01; DRB4*01:03; DRB5*02:02	DQA*01:02/03; DQB*03:01/05:02	1/0	0/0	1/0	0/0	0/3	0/3	0/1	0/1
	6	DRB1*01:01, 11:01; DRB3*02:02	DQA*01:01/05; DQB*03:01/05:01	1/1	1/1	1/1	1/1	0/1	0/1	0/1	0/0
	7	DRB1*03:01, 04:01; DRB3*01:01; DRB4*01:03	DQA*03/05; DQB*02/03:01	1/1	0/2	1/2	1/1	0/3	0/1	0/1	1/0
	8	DRB1*04:01, 15:01; DRB4*01:03; DRB5*01:01	DQA*01:02/03; DQB*03:01/06:02	1/1	0/0	1/0	0/0	1/3	0/1	0/2	0/0
	9	DRB1*03:01, 11:01; DRB3*01:01, 02:02	DQA*05; DQB*02/03:01	0/2	1/2	0/2	1/1	0/1	0/2	0/0	1/0
	10	DRB1*03:01, 04:01; DRB3*01:01; DRB4*01:03	DQA*03/05; DQB*02/03:01	1/1	0/2	1/2	1/1	0/3	0/1	0/1	1/0
	11	DRB1*04:01, 07:01; DRB4*01:03	DQA*02:01/03; DQB*03:01/03:03	2/0	1/1	1/0	1/1	0/2	0/3	0/2	0/0
HC	1	DRB1*04:01, 04:04; DRB4*01:03	DQA*03:01/03:03; DQB*03:01/03:02	1/0	0/1	1/0	0/0	0/3	0/1	0/1	0/1
	2	DRB1*04:01, 11:04; DRB3*02:02; DRB4*01:03	DQA*03:01/05:05; DQB*03:01	1/1	0/0	1/1	0/0	0/2	0/0	0/2	0/0
	3	DRB1*03:01, 11:04; DRB3*01:01, 02:02	DQA*05:01/05:05; DQB*02:01/03:01	0/1	1/2	0/2	1/1	0/1	0/2	0/1	1/0
	4	DRB1*07:01, 15:01; DRB4*01:03; DRB5*01:01	DQA*01:02/02:01; DQB*03:03/06:02	1/1	1/1	0/0	1/1	1/1	0/3	0/3	0/0
	5	DRB1*03:01, 13:01; DRB3*01:01	DQA*01:03/05:01; DQB*02:01/06:03	0/3	0/2	0/3	0/1	0/1	0/1	0/1	1/0
	6	DRB1*01:01, 03:01; DRB3*01:01	DQA*01:01/05:01; DQB*05:01/02:01	1/1	0/2	1/2	0/1	0/2	0/1	0/1	1/0
	7	DRB1*11:04, 13:02; DRB3*02:02, 03:01	DQA*01:02/05:05; DQB*03:01/06:04	0/2	0/0	0/1	0/0	0/0	0/2	0/2	0/0
	8	DRB1*13:01, 13:02; DRB3*02:02, 03:01	DQA*01:02/01:03; DQB*06:03/06:04	0/3	0/0	0/2	0/0	0/0	0/2	0/1	0/0
	9	DRB1*03:01; DRB3*01:01	DQA*05:01; DQB*02:01	0/1	0/2	0/2	0/1	0/1	0/1	0/0	1/0
	10	DRB1*15:01; DRB5*01:01	DQA*01:02; DQB*06:02	0/1	0/0	0/0	0/0	1/0	0/3	0/2	0/0
	11	DRB1*03:01, 13:01; DRB3*01:01	DQA*01:03/05:01; DQB*02:01/06:03	0/3	0/2	0/3	0/1	0/1	0/1	0/1	1/0
MassSpec	1	DRB1*04:02, 04:04; DRB4*01:01, 01:03		0/1						0/2	
	2	DRB1*13:01, *13:03*; DRB3*01:01, 02:02		0/3						0/1	
	3	DRB1*01:01, 14:01; DRB3*02:02		1/1						0/1	
	4	DRB1*04:02, 04:04; DRB4*01:03		0/1						0/2	
	5	DRB1*03:01, 07:01; DRB3*01:01; DRB4*01:01		1/1						0/2	

The number of predicted strong and medium binding (strong/medium), unique 10-mer peptides is indicated for each 22-mer peptide used. RA, rheumatoid arthritis patients; HC, healthy controls; MassSpec, donors used in peptide elution experiments.

The CH3-derived peptide H216–237 that was predicted not to bind to the HLA of the donors was included as a control. Human immature monocyte-derived dendritic cells (moDC) were incubated with the peptides, matured and HLA-DR/antigen complexes were affinity purified, followed by mass spectrometry of eluted peptides. moDC from 4 out of 5 (80%) tested donors presented the VH2-derived H41–62 peptide and 1 out of 4 (25%) presented the VL1-derived L18–39 peptide (**Table 3**). In contrast, the control samples in which the predicted non-binding H216–237 peptide or no peptides were added, showed no adalimumab-derived sequences in the mass spectrometry analyses (0/3 and 0/5, respectively), indicating that the method is specific and that peptide detection in mass spectrometry is not due to carry-over of non-bound peptides in the isolates. Interestingly, the HLA-DR–eluted peptides were present in multiple truncated forms, demonstrating that the adalimumab-derived peptides were subjected to peptidase processing (**Table 3**).

**Table 3:** Analysis of adalimumab-derived, HLA-DR-bound peptides by mass spectrometry.

Donor	H41-62	L18-39	H216-237 (predicted NBC)	No peptide
	PGKGLEWVSAITWNSGHIDYAD	RVTITCRASQGIRNYLAWYQQK	DKKVEPKSCDKTHTCPPCPAPE	
1	PGKGLEWVSAITWNSGHIDYAD	–	n.t.	–
	PGKGLEWVSAITWNSGHIDY			
	PGKGLEWVSAITWNSGHID			
	PGKGLEWVSAITWNSGHI			
	GKGLEWVSAITWNSGHIDYAD			
2	PGKGLEWVSAITWNSGHIDYAD	RASQGIRNYLAWYQQK	n.t.	–
	PGKGLEWVSAITWNSGHIDYA	ASQGIRNYLAWYQQK		
	PGKGLEWVSAITWNSGHIDY	ASQGIRNYLAWYQQ		
	PGKGLEWVSAITWNSGHID	SQGIRNYLAWYQQK		
	PGKGLEWVSAITWNSGH			
	PGKGLEWVSAITWNSG			
	GKGLEWVSAITWNSGHID			
	KGLEWVSAITWNSGHID			
	GLEWVSAITWNSGHID			
	GLEWVSAITWNSGHI			
3	PGKGLEWVSAITWNSGHIDYAD	–	–	–
	PGKGLEWVSAITWNSGHIDY			
	PGKGLEWVSAITWNSGHID			
	KGLEWVSAITWNSGHID			
	GLEWVSAITWNSGHID			
	GLEWVSAITWNSGHI			
4	PGKGLEWVSAITWNSGHIDYAD	–	–	–
	PGKGLEWVSAITWNSGHID			
5	–	n.t.	–	–

moDCs were derived from 5 healthy donors, incubated with indicated peptides during maturation and HLA-DR-bound peptides were subsequently purified and analysed using mass spectrometry. Top sequences represent original peptides used, and all peptides containing entire original sequence or its fragment are listed per donor N.t., not tested. H216–237 was used as a non-binding control (NBC) based on the prediction.

### Adalimumab-derived peptides induce CD4 T cell responses

**Figure 1:**
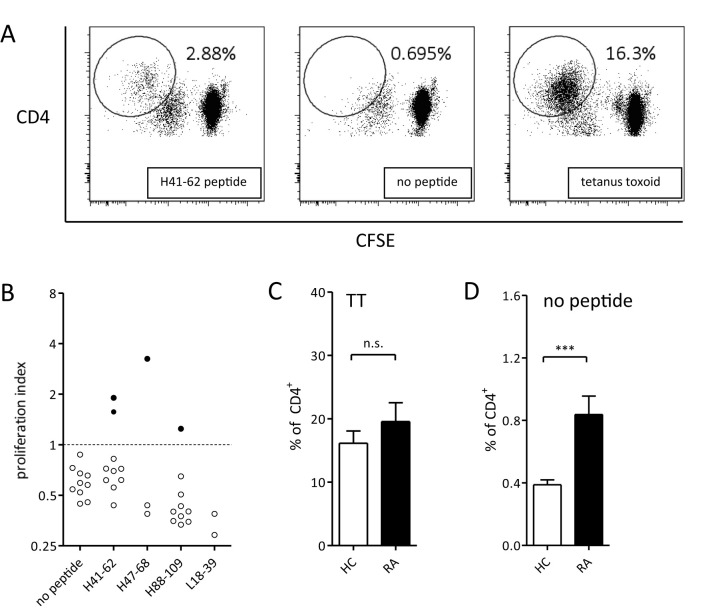
CD4 T cell responses of RA patients to adalimumab peptides and tetanus toxoid. **(A)** CD4 T cell proliferation after 14 days of PBMC culture with adalimumab-derived peptide (left), no peptide (middle), or tetanus toxoid (right). Representative plots from one donor out of eleven are shown. Antigen-specific T cells are gated as CD4hiCFSElow. **(B)** Summary of overall CD4 T cell response against adalimumab-derived peptides in one representative patient. Data are shown as proliferation index, calculated as fold increase in proliferation over mean + 3*SD of control wells (cut-off value) not containing any peptide. Every dot represents proliferation in a single well. Filled dots represent proliferation above, and open below, the cutoff. **(C, D)** CD4 T cell proliferation (mean ± SEM) of all patients (n=11) and healthy control donors (n=11) in response to tetanus toxoid **(C)** or medium only **(D)**. ***p<0.001 in unpaired t-test, n.s. - not statistically significant.

**Figure 2:**
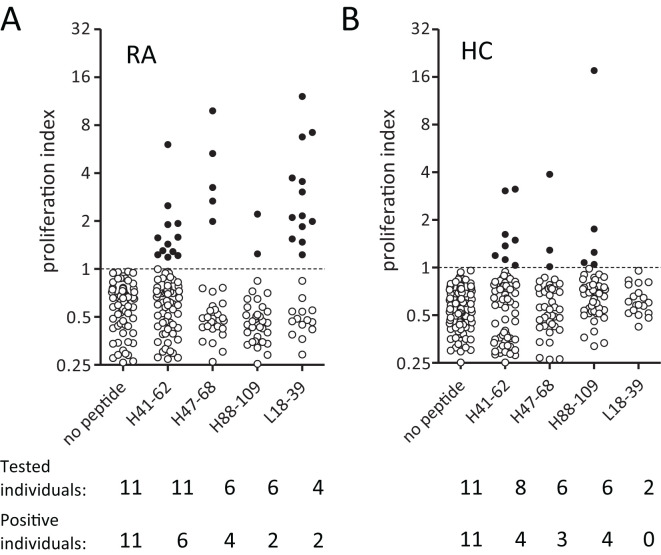
RA patients and healthy donors show CD4 T cell reactivity towards Epibase™-predicted HLA class II binding peptides from adalimumab. **(A, B)** Overall CD4 T cell proliferation after 14 days in PBMC cultured against different adalimumab-derived peptides in 11 RA patients **(A)** and 11 haplotype-matched healthy donors **(B)**. Every dot represents proliferation in a single well. Filled dots represent proliferation above, and open below, the cutoff value.

To investigate whether predicted HLA II epitopes of adalimumab can activate T cells, 11 adalimumab-experienced RA patients were analyzed for *in vitro* CD4 T cell reactivity to adalimumab peptides in PBMC co-cultures (**Figures 1A, B**). The cells were incubated with adalimumab sequence-derived or control peptides for 14 days. Peptides were derived from the variable regions, with preferential use of those that exhibited the strongest HLA-DR and HLA-DQ binding predictions when patient material was limited (**Table 2**). CD4 T cells proliferated to tetanus toxoid (**Figure 1A**, right), while non-specific proliferation in the absence of peptide was low (**Figure 1A**, middle). As expected, CD4 proliferation towards adalimumab peptides (**Figure 1A**, left) was much less than to tetanus toxoid, as the latter contains a multitude of epitopes, but CD4 T cell responses exceeding the mean proliferation + 3*SD of control wells (no peptide) could still be detected against several adalimumab-specific peptides in RA patients (**Figures 1B**, **2A**).

CD4 T cell responses were compared between 11 RA patients and 11 healthy donors that were HLA-typed and matched for most HLA alleles (**Table 2**). Tetanus-specific CD4 T cell responses showed no significant differences between RA patients and healthy controls (**Figure 1C**), demonstrating absence of generalized immune suppression in RA. Proliferation of T cells in absence of external antigen was significantly higher in the RA patient group (**Figure 1D**), indicating that non-specific proliferation was elevated in RA patients.

In line with a previous report (25), healthy donors could mount CD4 T cell reactivity to specific adalimumab-derived peptides (**Figure 2B**). RA patients did not respond with a noticeably higher overall frequency to adalimumab-peptides (**Figures 2A, B**, tested individuals and positive individuals). However, it’s worth noticing that unexposed donors appeared to react more frequently to the H88–109 peptide (5 responses in 4/6 healthy donors versus 2 responses in 2/6 RA patients), and that the response to L18–39 peptide was observed only in RA patients and at a relatively high frequency (suggesting a potential role in driving anti-adalimumab immunity.

As CD4 T cell responses are important for the generation of high-affinity IgG antibodies, the ability to mount anti-adalimumab T cell responses was compared to actual formation of anti-adalimumab antibodies in RA patients. CD4 T cell responses to adalimumab-derived peptides were detected in both antibody-forming and non-antibody-forming RA patients (**Table 4**). More patients mounted an anti-adalimumab CD4 T cell response than AAA (63.6% versus 45.5%). Importantly, all RA patients that developed AAA showed CD4 T cell reactivity to at least some of adalimumab-derived peptides. Altogether these results show that most adalimumab-treated RA-patients analyzed here have CD4 T cells recognizing adalimumab peptides. The presence of the CD4 T cell reactivity may be a permissive step in antibody formation process in exposed individuals, as emphasized by the fact that in this group of patients T cell reactivity against the therapeutic protein always accompanied AAA responses.

**Table 4:** Antibody formation and immunomodulators.

Patient	Antibody formation	Number of DMARD	MTX use	CD4 T cell responses
1	++	1	yes	++
2	–	6	yes	–
3	–	9	yes	++
4	++	7	yes	++
5	–	4	yes	–
6	–	2	yes	–
7	–	4	yes	++
8	–	3	yes	–
9	++	1	yes	++
10	++	5	yes	++
11	++	6	Yes	++
	% Ab formers	Average	% MTX	% Responders
	45.5%	4.4	100.0%	63.6%

The number of DMARD used are indicated at the time of blood donation. DMARD, disease-modifying anti-rheumatic drugs; MTX, methotrexate.

## Discussion

The frequent occurrence of high affinity IgG1 and IgG4 antibodies against adalimumab in treated patients, strongly point to CD4 T cell involvement. Here, we show that the variable regions of adalimumab contain MHC class II binding epitopes that indeed elicit CD4 T cell responses in patients. This is in line with the unique, thus foreign nature of the CDRs in the variable region and consistent with previous reports pointing towards the CDRs as drivers of immunogenicity towards therapeutic antibodies (24). It should be noted however, that reactivity against germline epitopes may still occur in individuals that show germline differences to adalimumab, as limited sequence variation does occur in the human population (33). So far, CD4 T cell responses specific for the immunoglobulin constant regions could not be demonstrated in humans (34). Polyvalent IgG has been shown to induce CD4+Foxp3+ Treg cells, but the mechanism is proposed to be driven by anti-idiotype responses (35), potentially in the context of parallel dominant germline peptide presentation. Many of the epitopes predicted in adalimumab can - according to the model - be presented by multiple HLA alleles and together cover major HLA allotypes, suggesting that most people can present adalimumab-derived peptides via HLA II and induce CD4 T cell reactivity against adalimumab.

*In vitro* evaluation of epitope prediction is essential, as in-silico methods are designed primarily with biding affinity in mind, which leads to over-prediction. Common pitfalls originate in the insufficient understanding and incorporation of antigen processing and presentation processes into the prediction model, as well as the inability to distinguish effector and tolerogenic epitopes. Mass spectrometry analysis of the HLA-DR-eluted peptides, although may not directly overcome those pitfalls, confirmed that presentation of variable region peptides by human moDC is possible. Ideally, this would have been done using patient-derived moDCs, accounting for possible differences in antigen processing, and whole antibody to engage classical processing pathway. With current experimental design, peptides can be loaded onto the HLA II molecules either directly on the surface of the APC or in the antigen loading compartment upon endocytosis. In our experiments various truncated forms of peptides were eluted from HLA II, demonstrating that they had, at least in part, been subject to peptidase activity before or after loading onto HLA II. This type of activity is commonly observed in the endosomes (36, 37), but extracellular processing cannot be excluded.

Qualitative analysis revealed CD4 T cell responses to multiple peptides that derive from regions previously shown to contain T cell epitopes in healthy donors (27, 38) except for the L18–39 peptide which did not produce any T cell response (despite strong binding signal). We did not observe the enhanced signal with H88–109 peptide, corresponding to the AH91–110 peptide region, reported in both publications. CD4 T cell responses to therapeutic antibodies have been generally challenging to identify. In healthy donors the frequency was estimated to be below 1 cell per million CD4 T cells for a variety of therapeutic antibodies with proven immunogenicity (25). For common vaccine antigens, average peptide-specific naïve CD4 T cells frequencies have been reported to be below 10 cells per million (39, 40). In vaccinated individuals, the specific frequency was increased 60 to 200-fold in the CD4 memory T cell compartment (40). In our assay, with 1 million PBMCs per well, which amounts to between 200,000 and 600,000 CD4 T cells per well, multiple positive wells would point towards an adalimumab-specific T cell frequency that matches classical memory response. Therefore, it is surprising that the number of positive wells in donors responding to adalimumab peptides is generally low (between 10-20%) and comparable between exposed patients and unexposed subjects. One of the reasons may be an underestimation of the T cell reactivity due to autoimmune background of RA patients and the elevated background signal that we observed. Alternatively, adalimumab-specific CD4 T cells may localize to peripheral tissues, especially in RA patients who show ectopic germinal center formation (41). Finally, some of the observed reactivities in both healthy subjects and patients can derive from the same naïve pool, and the peptides driving the anti-drug response can be rare and exclusive to certain HLA alleles. In line with this assumption, L18–39 peptide generated multiple positive wells for each responding patient, which is more fitting of the classical memory response. However, in this case, only two healthy donors were analyzed for the presence of peptide-specific T cells, making it difficult to unequivocally recognize the enhanced presence of these T cells in adalimumab-experienced patients. Overall, despite good HLA-DRB1 coverage, higher sample numbers for both RA patients and healthy donors would benefit this study and help to better understand the implications of the observed differences and similarities between the two groups. In addition, sorting the cells into naïve and memory subsets prior to stimulation could help unequivocally determine where the anti-adalimumab reactivity originates.

Despite these drawbacks, L18–39 showed the highest potential of all tested peptides to play an important role in anti-adalimumab immunity. It did not induce any response in healthy donors and have not been previously identified in healthy donor studies.

The finding that all antibody-forming patients showed CD4 T cell reactivity towards adalimumab-derived peptides supports the hypothesis that CD4 T cell involvement is necessary for AAA development. As not all patients with anti-adalimumab CD4 T cell reactivity developed AAA, our data also indicate that CD4 reactivity is not the limiting parameter and that other factors are involved. These include effects of co-medication, as well as potential involvement of regulatory T and B cells. It is also important to stress that the assay used to determine AAA levels in patients doesn’t allow to distinguish between true non-antibody formers and low-level antibody formers where the AAA levels are insufficient to neutralize the circulating drug and therefore some functional non-antibody formers could be generating AAA to a lower extent. However, this type of antibody production is less relevant from the clinical standpoint, maintaining functional circulating drug.

We had previously shown that B cell reactivity, and therefore the specificity of the produced antibodies, is directed towards the idiotype of adalimumab (16). Our data now show that the variable domains of adalimumab also elicit CD4 T cell responses, which has implications for development of therapeutic antibodies in general. Identification of CD4 T cell epitopes in biological agents and subsequent modification of these epitopes have been suggested as one way to reduce immunogenicity of the therapeutic protein (24, 42). A recent study in macaques has shown that combined elimination of B cell epitopes, T cell epitopes and aggregation-prone regions can successfully reduce immunogenic potential of adalimumab (43). An additional option may be to devise treatment strategies that target undesired CD4 T cell responses against biologicals. Induction of specific regulatory T cells or CD4 T cells that do not support B cell differentiation and antibody formation may prevent ADA formation even when patients require prolonged treatment. This epitope-based approach had been reviewed extensively (44). Further research into de-immunization of the protein therapeutics, co-medication with immunomodulating drugs (35), adaptation of treatment dosage and schedules (45), and tolerance-inducing T cell therapies (46) may pave the way for effective biologicals therapy while avoiding undesired drug immunogenicity.

## Materials and methods

### Patients and samples

Heparinized peripheral blood was obtained from 11 RA patients (**Table 4**) and 11 healthy donors. Patients were recruited from a cohort of 121 RA patients consecutively treated with adalimumab at the Department of Rheumatology, Reade, and the Academic Medical Centre in Amsterdam (The Netherlands) and carefully monitored longitudinally for adalimumab trough levels, AAA-formation and clinical efficacy, as described (12). Patients were treated with concomitant disease modifying anti-rheumatic drugs (DMARD). Patients received an adalimumab dose of 40 mg subcutaneously every other week, increasing to weekly doses in patients with insufficient response. Samples were collected at various time points after at least 1 year of treatment with adalimumab. Healthy donors recruited from the internal blood donation network at Sanquin Blood Supply Foundation, Amsterdam, were HLA-typed and matched as closely as possible for most HLA alleles. The HLA-DRB1 allele distribution in both study groups against population distribution of Leiden, Netherlands is shown in **Supplementary Figure S1**. The Medical Research and Ethics Committee of the Academic Medical Centre Amsterdam approved the study, and all study participants gave written informed consent.

### HLA-typing of patients and healthy donors

DNA was isolated from peripheral blood with QIAmp DNA Blood Mini Kit (Qiagen). HLA genotyping was performed for the HLA-DRB1, DRB3, DRB4, DRB5, DQB1 and DQA1 alleles with high resolution using sequence-specific primers (Sanquin Diagnostic Services).

### Measuring anti-adalimumab antibodies in antigen binding test

AAA titers were measured in serum samples of patients as described (47, 48). Briefly, antibodies were captured from plasma samples on protein A Sepharose and AAA were detected with ^125^I labelled adalimumab F(ab’)_2_ diluted in Freeze buffer (Sanquin). Results were expressed in arbitrary units (AU) in reference to serum standards. Samples were considered positive for AAA if titers were greater than 12 AU/ml. Patients were tested at different time points within a 24-month period after start of adalimumab treatment and were considered antibody formers when tested positive on at least one occasion. Samples for antibody detection were drawn just prior to the next adalimumab injection when trough levels of adalimumab are expected.

### CD4 T cell epitope prediction

Potential CD4 T cell epitopes were predicted *in silico* with the Epibase™ v.2 prediction tool (Lonza) (49). This platform analyses the HLA binding specificities of all possible, overlapping, 10-mer peptides by sliding a window of 10 amino acids throughout the target sequence and applying a scoring function on each 10-mer peptide sequence (50–52). Profiling is done at the allotype level for 20 DRB1, 7 DRB3/4/5, 14 DQ and 7 DP HLA class II molecules. The resulting free energy binding values are converted to dissociation constants for each of the HLA class II/peptide complexes. This allows classification of the peptides as strong (Kd < 0.100 μM), medium (0.100 μM ≤ Kd ≤ 0.799 μM) and weak to non-binders (0.799 μM ≤ Kd).

### Peptide synthesis

All peptides were synthesized with standard Fmoc-SPPS (9-fluorenylmethyloxycarbonyl solid-phase peptide synthesis) chemistry on a SyroII synthesizer (MultiSynTech) using PyBop as an activator. Control and purification of peptides was performed on HPLC with a Waters reversed phase C18 column using a water-acetonitrile gradient acidified with 0.05% trifluoroacetic acid. Final purity was determined with a liquid chromatography electrospray mass spectrometer. All peptides were soluble and present as monomers in solution. In addition, all peptides were controlled for signs of gellification, cloudiness or particulates by visual inspection and upon thawing the aliquots and were additionally spun down to eliminate potential aggregates.

### Dendritic cells generation, peptide elution and mass spectrometry

Monocytes were isolated by Elutra (Gambro) from fresh aphaeresis material of healthy volunteers (53). Monocytes were cultured at 1×10^6^ cells/ml in Nunclon Delta Surface flasks (Nunc) in serum-free CellGro DC Medium supplemented with 800 IU/ml IL-4, 1000 IU/ml GM-CSF (CellGenix), 100 IU/ml penicillin and 100 μg/ml streptomycin. After 6 days, immature dendritic cells were washed and re-plated in CellGro medium at a concentration of 2.5×10^6^ cells/ml in a final volume of 2 ml and incubated with adalimumab-derived peptides at a concentration of 10 μg/ml for 4 hours and matured with 2.5 μg/ml of MPLA (Sigma-Aldrich) and 1000 U/ml IFNγ (Immukine, Boehringer Ingelheim) for 24 hours before harvesting (54). Isolation of the MHC class II/peptide complexes, peptide elution and purification, and mass spectrometry measurements were performed as described previously (55). Briefly, cell pellets were lysed in 50 mM Tris pH 7.0 containing 4% Igepal CA-630 (Sigma) and HLA-DR was purified by affinity chromatography with L243 antibody coupled to CNBr Sepharose 4B (Amersham Biosciences) in the presence of protease inhibitors (Complete Protease Inhibitor Mixture Tablet, Roche Diagnostics GmbH). Peptides were eluted by incubation with 10% acetic acid for 15 min at 70°C and purified using a C18 ZipTip (Millipore). Eluted peptides were separated using a reversed-phase C18 column (Nanoseparations) and sprayed directly into the LTQ Orbitrap XL mass spectrometer (Thermo Fisher Scientific) for measurement. Adalimumab-specific peptides were identified using a Sequest search algorithm against UniprotKB, a non-redundant protein database extended by addition of the adalimumab sequence (56).

### Isolation of PBMC, T cell proliferation assays and flow cytometric analysis

Peripheral blood mononuclear cells (PBMC) were isolated using Lymphoprep (Axis-Shield), stained with 0.5 μM CFDA-SE (Invitrogen) for 15 minutes at room temperature and cultured, after extensive washing in Iscove’s Modified Dulbecco’s Medium (Lonza) containing 5% Human Serum (Sanquin), 100 U/ml penicillin and 100 µg/ml streptomycin. 1×10^6^ PBMCs/well were cultured for 14 days (37°C,5% CO_2_) with 10 μg/ml adalimumab peptides or 5 μg/ml tetanus toxoid (TT, Statens Serum Institute) in 1ml of medium in a 24-well plate format (Corning Costar), with 5–10 wells dedicated to each peptide based on the total number of cells available Media was supplemented when needed based on the pH indicator by replacing 50% of the well media with fresh one.

Peptides: H41-62 (PGKGLEWVSAITWNSGHIDYAD) – located in CDR-H2, H47-68 (WVSAITWNSGHIDYADSVEGRF) - located in CDR-H2, H88-109 (AEDTAVYYCAKVSYLSTASSLD) – located in CDR-H3, L18-39 (RVTITCRASQGIRNYLAWYQQK) – located in CDR-L1, H216-237 (DKKVEPKSCDKTHTCPPCPAPE) – located in CH3. H and L indicate heavy or light chain and the numbers refer to the position in the original sequence of adalimumab. To detect low proliferation frequencies ten wells were plated per condition (two for tetanus toxoid). Harvested cells were labelled with anti-CD4-APC (BD Biosciences), measured on the LSRII flow cytometer (BD), and analyzed with FlowJo (Tree star) analysis software. Results of CD4 T cell proliferation were normalized using the mean proliferation + 3*SD of the control wells (no peptide) as reference and cutoff value.

Multiple control experiments were performed to determine the ability of various stimulating factors to proliferate CD4 T cells in our assay. Those factors included protein antigens: PPD, TT (Statens Serum Institute), peptide mix: CMV pp65 (Miltenyi biotec) and TT-derived peptides at a concentration of 5 μg/ml (**Supplementary Figure S2**).

### Statistical analysis

Statistical differences were determined using Prism 9 (GraphPad).

## Data Availability

The original contributions presented in the study are included in the article/supplementary material. Further inquiries can be directed to the corresponding author.

## Supplementary material

Supplementary Figure 1 The HLA-DRB1 allele distribution in healthy donors (n=11) and RA patients (n=11) included in this study, as well as control dataset of population of Leiden, Netherlands (n=1305) extracted from http://www.allelefrequencies.net database.

Supplementary Figure 2 Sensitivity validation of the main T cell proliferation assay. PBMCs from two healthy donors were used – donor 1: conditions 1-4 (4 wells per condition), and donor 2: conditions 5-7 (3 wells per condition). The proliferative response was shown using whole protein antigens (PPD, TT), peptide pool (CMV pp65), and selected TT peptides.

## References

[1] Tracey D Klareskog L Sasso EH Salfeld JG Tak PP. Tumor necrosis factor antagonist mechanisms of action: a comprehensive review. Pharmacol Ther. (2008) 117:244–79. doi: 10.1016/j.pharmthera.2007.10.001 18155297

[2] Saxne T Palladino MA Heinegård D Talal N Wollheim FA. Detection of tumor necrosis factor alpha but not tumor necrosis factor beta in rheumatoid arthritis synovial fluid and serum. Arthritis Rheum. (1988) 31:1041–5. doi: 10.1002/art.1780310816 3136775

[3] Brennan FM Chantry D Jackson A Maini R Feldmann M. Inhibitory effect of TNF alpha antibodies on synovial cell interleukin-1 production in rheumatoid arthritis. Lancet. (1989) 2:244–7. doi: 10.1016/S0140-6736(89)90430-3 2569055

[4] Keffer J Probert L Cazlaris H Georgopoulos S Kaslaris E Kioussis D. Transgenic mice expressing human tumour necrosis factor: a predictive genetic model of arthritis. EMBO J. (1991) 10:4025–31. doi: 10.1002/j.1460-2075.1991.tb04978.x PMC453150 1721867

[5] Haraoui B. Differentiating the efficacy of the tumor necrosis factor inhibitors. Semin Arthritis Rheum. (2005) 34:7–11. doi: 10.1016/j.semarthrit.2005.01.003 15852248

[6] Elliott MJ Maini RN Feldmann M Kalden JR Antoni C Smolen JS. Randomised double-blind comparison of chimeric monoclonal antibody to tumour necrosis factor alpha (cA2) versus placebo in rheumatoid arthritis. Lancet. (1994) 344:1105–10. doi: 10.1016/S0140-6736(94)90628-9 7934491

[7] Weinblatt ME Kremer JM Bankhurst AD Bulpitt KJ Fleischmann RM Fox RI. A trial of etanercept, a recombinant tumor necrosis factor receptor:Fc fusion protein, in patients with rheumatoid arthritis receiving methotrexate. N Engl J Med. (1999) 340:253–9. doi: 10.1056/NEJM199901283400401 9920948

[8] Lipsky PE van der Heijde DM St Clair EW Furst DE Breedveld FC Kalden JR. Infliximab and methotrexate in the treatment of rheumatoid arthritis. Anti-Tumor Necrosis Factor Trial in Rheumatoid Arthritis with Concomitant Therapy Study Group. N Engl J Med. (2000) 343:1594–602. doi: 10.1056/NEJM200011303432202 11096166

[9] Weinblatt ME Keystone EC Furst DE Moreland LW Weisman MH Birbara CA. Adalimumab, a fully human anti-tumor necrosis factor alpha monoclonal antibody, for the treatment of rheumatoid arthritis in patients taking concomitant methotrexate: the ARMADA trial. Arthritis Rheum. (2003) 48:35–45. doi: 10.1002/art.10697 12528101

[10] Olsen NJ Stein CM. New drugs for rheumatoid arthritis. N Engl J Med. (2004) 350:2167–79. doi: 10.1056/NEJMra032906 15152062

[11] Wolbink GJ Vis M Lems W Voskuyl AE de Groot E Nurmohamed MT. Development of antiinfliximab antibodies and relationship to clinical response in patients with rheumatoid arthritis. Arthritis Rheum. (2006) 54:711–5. doi: 10.1002/art.21671 16508927

[12] Bartelds GM Wijbrandts CA Nurmohamed MT Stapel S Lems WF Aarden L. Clinical response to adalimumab: relationship to anti-adalimumab antibodies and serum adalimumab concentrations in rheumatoid arthritis. Ann Rheum Dis. (2007) 66:921–6. doi: 10.1136/ard.2006.065615 PMC1955110 17301106

[13] van Kuijk AWR de Groot M Stapel SO Dijkmans BAC Wolbink GJ Tak PP. Relationship between the clinical response to adalimumab treatment and serum levels of adalimumab and anti-adalimumab antibodies in patients with psoriatic arthritis. Ann Rheum Dis. (2010) 69:624–5. doi: 10.1136/ard.2009.108787 20223840

[14] Bartelds GM Krieckaert CLM Nurmohamed MT van Schouwenburg PA Lems WF Twisk JWR. Development of antidrug antibodies against adalimumab and association with disease activity and treatment failure during long-term follow-up. JAMA: J Am Med Assoc. (2011) 305:1460–8. doi: 10.1001/jama.2011.406 21486979

[15] van Schouwenburg PA Krieckaert CL Rispens T Aarden L Wolbink GJ Wouters D. Long-term measurement of anti-adalimumab using pH-shift-anti-idiotype antigen binding test shows predictive value and transient antibody formation. Ann Rheum Dis. (2013) 72:1680–6. doi: 10.1136/annrheumdis-2012-202407 23300118

[16] van Schouwenburg PA van de Stadt LA de Jong RN van Buren EEL Kruithof S de Groot E. Adalimumab elicits a restricted anti-idiotypic antibody response in autoimmune patients resulting in functional neutralisation. Ann Rheum Dis. (2013) 72:104–9. doi: 10.1136/annrheumdis-2012-201445 22759910

[17] Radstake TRDJ Svenson M Eijsbouts AM van den Hoogen FHJ Enevold C van Riel PLCM. Formation of antibodies against infliximab and adalimumab strongly correlates with functional drug levels and clinical responses in rheumatoid arthritis. Ann Rheum Dis. (2009) 68:1739–45. doi: 10.1136/ard.2008.092833 19019895

[18] van de Putte LBA Atkins C Malaise M Sany J Russell AS van Riel PLCM. Efficacy and safety of adalimumab as monotherapy in patients with rheumatoid arthritis for whom previous disease modifying antirheumatic drug treatment has failed. Ann Rheum Dis. (2004) 63:508–16. doi: 10.1136/ard.2003.013052 PMC1755008 15082480

[19] van der Bijl AE Breedveld FC Antoni CE Kalden JR Kary S Burmester GR. An open-label pilot study of the effectiveness of adalimumab in patients with rheumatoid arthritis and previous infliximab treatment: relationship to reasons for failure and anti-infliximab antibody status. Clin Rheumatol. (2008) 27:1021–8. doi: 10.1007/s10067-008-0866-4 PMC2468311 18350329

[20] Maini RN Breedveld FC Kalden JR Smolen JS Davis D Macfarlane JD. Therapeutic efficacy of multiple intravenous infusions of anti-tumor necrosis factor alpha monoclonal antibody combined with low-dose weekly methotrexate in rheumatoid arthritis. Arthritis Rheum. (1998) 41:1552–63. doi: 10.1002/1529-0131(199809)41:9<1552::AID-ART5>3.0.CO;2-W 9751087

[21] Bendtzen K Geborek P Svenson M Larsson L Kapetanovic MC Saxne T. Individualized monitoring of drug bioavailability and immunogenicity in rheumatoid arthritis patients treated with the tumor necrosis factor alpha inhibitor infliximab. Arthritis Rheum. (2006) 54:3782–9. doi: 10.1002/art.22214 17133559

[22] Kay J Matteson EL Dasgupta B Nash P Durez P Hall S. Golimumab in patients with active rheumatoid arthritis despite treatment with methotrexate: a randomized, double-blind, placebo-controlled, dose-ranging study. Arthritis Rheum. (2008) 58:964–75. doi: 10.1002/art.23383 18383539

[23] Clark M. Antibody humanization: a case of the “Emperor’s new clothes”? Immunol Today. (2000) 21:397–402. doi: 10.1016/s0167-5699(00)01680-7 10916143

[24] Harding FA Stickler MM Razo J DuBridge RB. The immunogenicity of humanized and fully human antibodies: residual immunogenicity resides in the CDR regions. MAbs. (2010) 2:256–65. doi: 10.4161/mabs.2.3.11641 PMC2881252 20400861

[25] Delluc S Ravot G Maillere B. Quantitative analysis of the CD4 T-cell repertoire specific to therapeutic antibodies in healthy donors. FASEB J. (2011) 25:2040–8. doi: 10.1096/fj.10-173872 21368101

[26] Ito S Ikuno T Mishima M Yano M Hara T Kuramochi T. *In vitro* human helper T-cell assay to screen antibody drug candidates for immunogenicity. J Immunotoxicol. (2019) 16:125–32. doi: 10.1080/1547691X.2019.1604586 31179789

[27] Meunier S de Bourayne M Hamze M Azam A Correia E Menier C. Specificity of the T cell response to protein biopharmaceuticals. Front Immunol. (2020) 11:1550. doi: 10.3389/FIMMU.2020.01550 32793213 PMC7387651

[28] Lee MV Saad OM Wong S LaMar J Kamen L Ordonia B. Development of a semi-automated MHC-associated peptide proteomics (MAPPs) method using streptavidin bead-based immunoaffinity capture and nano LC-MS/MS to support immunogenicity risk assessment in drug development. Front Immunol. (2023) 14:1295285/FULL. doi: 10.3389/FIMMU.2023.1295285/FULL 38022649 PMC10667718

[29] Andrick BJ Schwab AI Cauley B O’Donnell LA Meng WS. Predicting hemagglutinin MHC-II ligand analogues in anti-TNFα Biologics: implications for immunogenicity of pharmaceutical proteins. PloS One. (2015) 10:e0135451. doi: 10.1371/journal.pone.0135451 26270649 PMC4536234

[30] Edwards JA Durant BM Jones DB Evans PR Smith JL. Differential expression of HLA class II antigens in fetal human spleen: relationship of HLA-DP, DQ, and DR to immunoglobulin expression. J Immunol. (1986) 137:490–7. doi: 10.4049/jimmunol.137.2.490 3522732

[31] Hauber I Gulle H Wolf HM Maris M Eggenbauer H Eibl MM. Molecular characterization of major histocompatibility complex class II gene expression and demonstration of antigen-specific T cell response indicate a new phenotype in class II-deficient patients. J Exp Med. (1995) 181:1411–23. doi: 10.1084/jem.181.4.1411 PMC2191976 7699327

[32] Sidney J Steen A Moore C Ngo S Chung J Peters B. Five HLA-DP molecules frequently expressed in the worldwide human population share a common HLA supertypic binding specificity. J Immunol. (2010) 184:2492–503. doi: 10.4049/jimmunol.0903655 PMC2935290 20139279

[33] Bartelds GM de Groot E Nurmohamed MT Hart MHL van Eede PH Wijbrandts CA. Surprising negative association between IgG1 allotype disparity and anti-adalimumab formation: a cohort study. Arthritis Res Ther. (2010) 12:R221. doi: 10.1186/ar3208 21187010 PMC3046534

[34] Eyerman MC Zhang X Wysocki LJ. T cell recognition and tolerance of antibody diversity. J Immunol. (1996) 157:1037–46. doi: 10.4049/jimmunol.157.3.1037 8757607

[35] Schmalzing M Behrens F Schwaneck EC Koehm M Greger G Gnann H. Does concomitant methotrexate confer clinical benefits in patients treated with prior biologic therapy? Analysis of data from a noninterventional study of rheumatoid arthritis patients initiating treatment with adalimumab. Med (United States). (2020) 99:E20201. doi: 10.1097/MD.0000000000020201 PMC7220320 32384515

[36] Larsen SL Pedersen LO Buus S Stryhn A. T cell responses affected by aminopeptidase N (CD13)-mediated trimming of major histocompatibility complex class II-bound peptides. J Exp Med. (1996) 184:183–9. doi: 10.1084/jem.184.1.183 PMC2192675 8691132

[37] Nelson CA Vidavsky I Viner NJ Gross ML Unanue ER. Amino-terminal trimming of peptides for presentation on major histocompatibility complex class II molecules. Proc Natl Acad Sci U.S.A. (1997) 94:628–33. doi: 10.1073/pnas.94.2.628 PMC19564 9012835

[38] Meunier S Hamze M Karle A de Bourayne M Gdoura A Spindeldreher S. Impact of human sequences in variable domains of therapeutic antibodies on the location of CD4 T-cell epitopes. Cell Mol Immunol. (2020) 17:656–8. doi: 10.1038/s41423-019-0304-3 PMC7264247 31659246

[39] Chu HH Moon JJ Takada K Pepper M Molitor JA Schacker TW. Positive selection optimizes the number and function of MHCII-restricted CD4+ T cell clones in the naive polyclonal repertoire. Proc Natl Acad Sci U.S.A. (2009) 106:11241–5. doi: 10.1073/pnas.0902015106 PMC2708705 19541603

[40] Kwok WW Tan V Gillette L Littell CT Soltis MA LaFond RB. Frequency of epitope-specific naive CD4(+) T cells correlates with immunodominance in the human memory repertoire. J Immunol. (2012) 188:2537–44. doi: 10.4049/jimmunol.1102190 PMC3997369 22327072

[41] Weyand CM Goronzy JJ. Ectopic germinal center formation in rheumatoid synovitis. Ann N Y Acad Sci. (2003) 987:140–9. doi: 10.1111/j.1749-6632.2003.tb06042.x 12727633

[42] Lu Z-J Deng S-J Huang D-G He Y Lei M Zhou L. Frontier of therapeutic antibody discovery: The challenges and how to face them. World J Biol Chem. (2012) 3:187–96. doi: 10.4331/wjbc.v3.i12.187 PMC3531614 23275803

[43] Kovalova N Boyles J Wen Y Witcher DR Brown-Augsburger PL Wroblewski VJ. Validation of a de-immunization strategy for monoclonal antibodies using cynomolgus macaque as a surrogate for human. Biopharm Drug Dispos. (2020) 41:111–25. doi: 10.1002/bdd.2222 32080869

[44] Schurgers E Wraith DC. Induction of tolerance to therapeutic proteins with antigen-processing independent T cell epitopes: controlling immune responses to biologics. Front Immunol. (2021) 12:742695. doi: 10.3389/fimmu.2021.742695 34567009 PMC8459012

[45] Benzaquen M Munshi M Bossart S Feldmeyer L Emelianov V Yawalkar N. Long-term dose optimization of adalimumab via dose spacing in patients with psoriasis. Bioengineering. (2022) 9(8):387. doi: 10.3390/bioengineering9080387 36004912 PMC9405054

[46] ten Brinke A Martinez-Llordella M Cools N Hilkens CMU Van Ham SM Sawitzki B. Ways forward for tolerance-inducing cellular therapies- An afactt perspective. Front Immunol. (2019) 10:181. doi: 10.3389/fimmu.2019.00181 30853957 PMC6395407

[47] Witteman AM Stapel SO Sjamsoedin DH Jansen HM Aalberse RC van der Zee JS. Fel d 1-specific IgG antibodies induced by natural exposure have blocking activity in skin tests. Int Arch Allergy Immunol. (1996) 109:369–75. doi: 10.1159/000237265 8634522

[48] Rispens T de Vrieze H de Groot E Wouters D Stapel S Wolbink GJ. Antibodies to constant domains of therapeutic monoclonal antibodies: anti-hinge antibodies in immunogenicity testing. J Immunol Methods. (2012) 375:93–9. doi: 10.1016/j.jim.2011.09.011 21986105

[49] Van Walle I Gansemans Y Parren PWHI Stas P Lasters I. Immunogenicity screening in protein drug development. Expert Opin Biol Ther. (2007) 7:405–18. doi: 10.1517/14712598.7.3.405 17309332

[50] Desmet J Wilson IA Joniau M De Maeyer M Lasters I. Computation of the binding of fully flexible peptides to proteins with flexible side chains. FASEB J. (1997) 11:164–72. doi: 10.1096/fasebj.11.2.9039959 9039959

[51] Desmet J Spriet J Lasters I. Fast and accurate side-chain topology and energy refinement (FASTER) as a new method for protein structure optimization. Proteins. (2002) 48:31–43. doi: 10.1002/prot.10131 12012335

[52] Desmet J Meersseman G Boutonnet N Pletinckx J De Clercq K Debulpaep M. Anchor profiles of HLA-specific peptides: analysis by a novel affinity scoring method and experimental validation. Proteins. (2005) 58:53–69. doi: 10.1002/prot.20302 15526297

[53] ten Brinke A Karsten ML Dieker MC Zwaginga JJ Vrielink H Marieke van Ham S. Generation of dendritic cells for immunotherapy is minimally impaired by granulocytes in the monocyte preparation. Immunobiology. (2006) 211:633–40. doi: 10.1016/j.imbio.2006.05.012 16920502

[54] Ten Brinke A Karsten ML Dieker MC Zwaginga JJ van Ham SM Jan J. The clinical grade maturation cocktail monophosphoryl lipid A plus IFNgamma generates monocyte-derived dendritic cells with the capacity to migrate and induce Th1 polarization. Vaccine. (2007) 25:7145–52. doi: 10.1016/j.vaccine.2007.07.031 17719152

[55] van Haren SD Herczenik E ten Brinke A Mertens K Voorberg J Meijer AB. HLA-DR-presented peptide repertoires derived from human monocyte-derived dendritic cells pulsed with blood coagulation factor VIII. Mol Cell Proteomics. (2011) 10:M110.002246. doi: 10.1074/mcp.M110.002246 PMC3108829 21467215

[56] The universal protein resource (UniProt) 2009. Nucleic Acids Res. (2009) 37:D169–74. doi: 10.1093/nar/gkn664 PMC2686606 18836194

