# Supplementary figures and images for: The TNFα-binding domain of the therapeutic antibody adalimumab elicits CD4 T-cell responses in rheumatoid arthritis patients
Source: Front Immunol. 2025 Jul 4;16:1549781. doi: 10.3389/fimmu.2025.1549781 (PMC12273444; doi:10.3389/fimmu.2025.1549781)

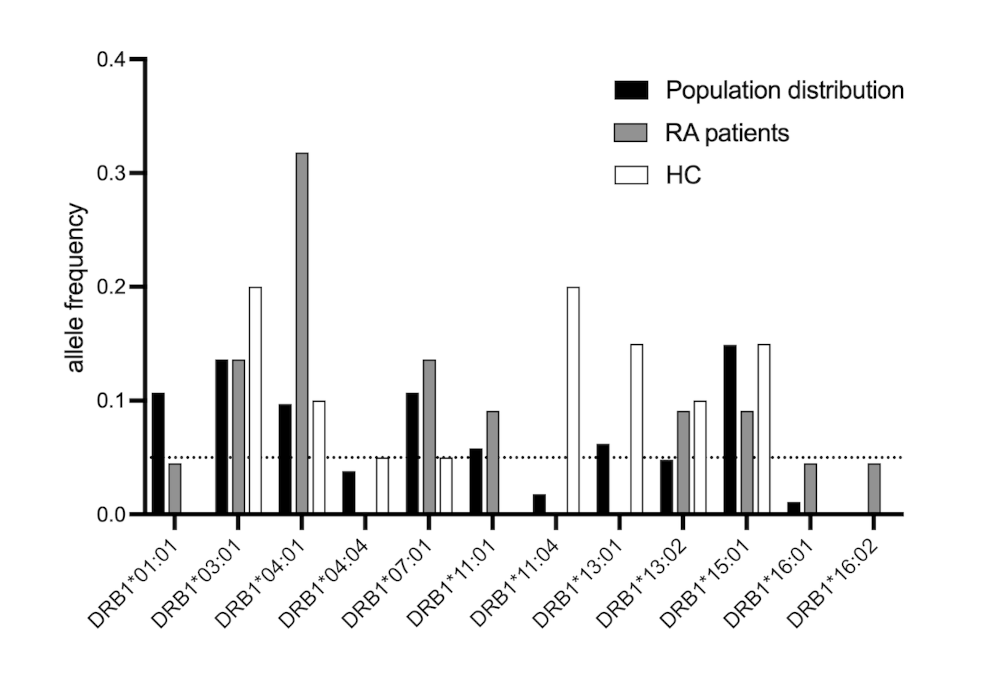

Supplement: Supplementary Figure 1 — The HLA-DRB1 allele distribution in healthy donors (n=11) and RA patients (n=11) included in this study, as well as control dataset of population of Leiden, Netherlands (n=1305) extracted from http://www.allelefrequencies.net database. [file Image1.tiff]

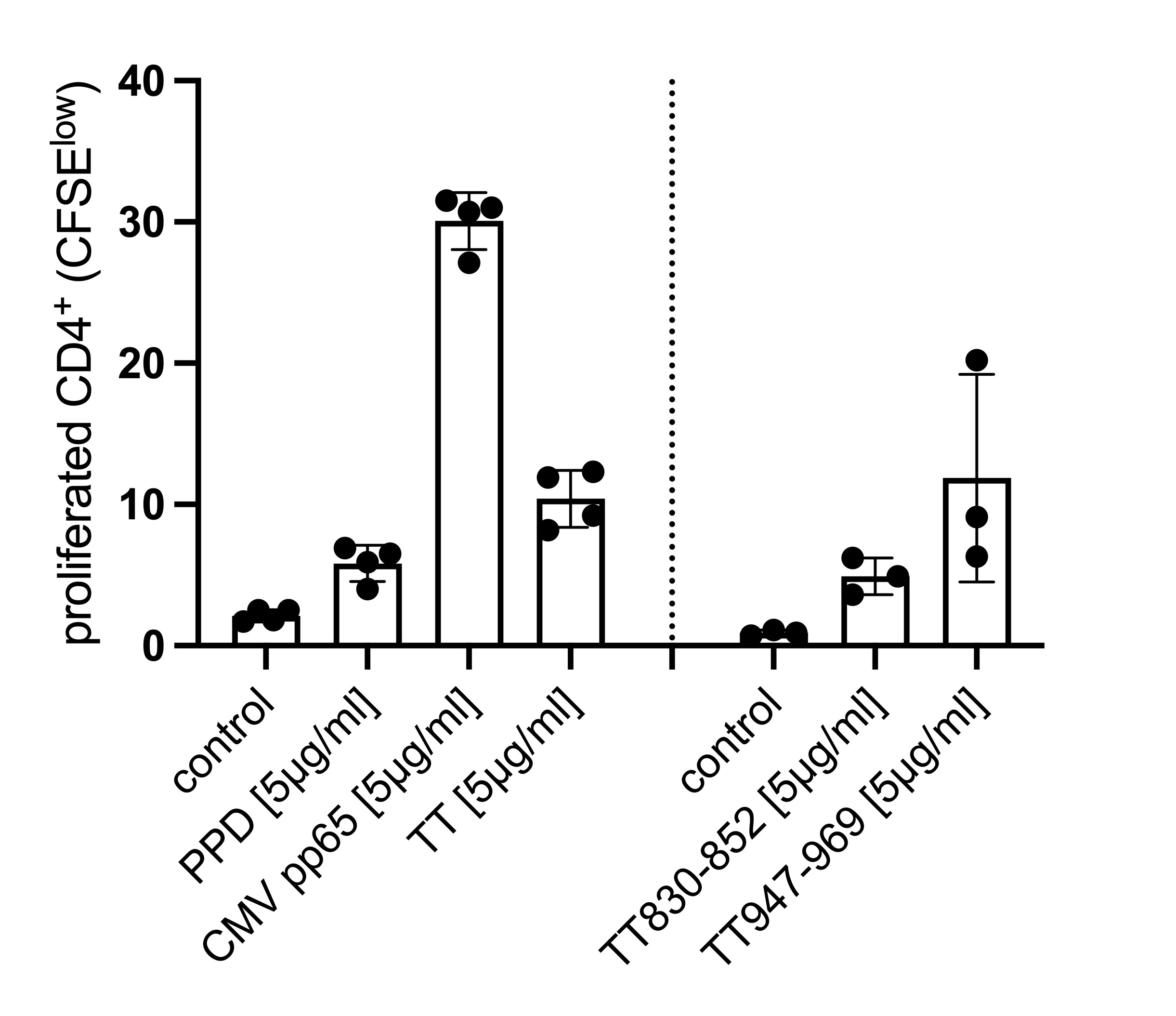

Supplement: Supplementary Figure 2 — Sensitivity validation of the main T cell proliferation assay. PBMCs from two healthy donors were used – donor 1: conditions 1-4 (4 wells per condition), and donor 2: conditions 5-7 (3 wells per condition). The proliferative response was shown using whole protein antigens (PPD, TT), peptide pool (CMV pp65), and selected TT peptides. [file Image2.jpeg]
